# The phototroph-specific β-hairpin structure of the γ subunit of F_o_F_1_-ATP synthase is important for efficient ATP synthesis of cyanobacteria

**DOI:** 10.1016/j.jbc.2021.101027

**Published:** 2021-07-31

**Authors:** Kumiko Kondo, Masayuki Izumi, Kosuke Inabe, Keisuke Yoshida, Mari Imashimizu, Toshiharu Suzuki, Toru Hisabori

**Affiliations:** Laboratory for Chemistry and Life Science, Tokyo Institute of Technology, Midori-ku, Yokohama, Japan

**Keywords:** F_o_F_1_, ATP synthase, ATPase, γ subunit, cyanobacteria, photosynthesis, *Synechocystis* sp. PCC 6803, *Thermosynechococcus elongatus* BP-1, LDAO, lauryl dimethylamine oxide, LMNG, lauryl maltose neopentyl glycol, PL, proteoliposome, *pmf*, proton motive force

## Abstract

The F_o_F_1_ synthase produces ATP from ADP and inorganic phosphate. The γ subunit of F_o_F_1_ ATP synthase in photosynthetic organisms, which is the rotor subunit of this enzyme, contains a characteristic β-hairpin structure. This structure is formed from an insertion sequence that has been conserved only in phototrophs. Using recombinant subcomplexes, we previously demonstrated that this region plays an essential role in the regulation of ATP hydrolysis activity, thereby functioning in controlling intracellular ATP levels in response to changes in the light environment. However, the role of this region in ATP synthesis has long remained an open question because its analysis requires the preparation of the whole F_o_F_1_ complex and a transmembrane proton-motive force. In this study, we successfully prepared proteoliposomes containing the entire F_o_F_1_ ATP synthase from a cyanobacterium, *Synechocystis* sp. PCC 6803, and measured ATP synthesis/hydrolysis and proton-translocating activities. The relatively simple genetic manipulation of *Synechocystis* enabled the biochemical investigation of the role of the β-hairpin structure of F_o_F_1_ ATP synthase and its activities. We further performed physiological analyses of *Synechocystis* mutant strains lacking the β-hairpin structure, which provided novel insights into the regulatory mechanisms of F_o_F_1_ ATP synthase in cyanobacteria *via* the phototroph-specific region of the γ subunit. Our results indicated that this structure critically contributes to ATP synthesis and suppresses ATP hydrolysis.

Photosynthetic organisms utilize F_o_F_1_ ATP synthase (F_o_F_1_) for a solar-to-chemical energy conversion system to produce ATP, the universal energy currency for cells. Under illumination, photosynthetic electron transport is activated, generating a proton electrochemical gradient (proton motive force, *pmf*) across the thylakoid membrane, which drives ATP synthesis (1, 2, 3, 4). In the dark, or when the *pmf* is insufficient, F_o_F_1_ hydrolyzes ATP as the reverse reaction and transports H^+^ to the lumen. Therefore, it is reasonable to assume that these organisms have evolved a unique mechanism for regulating F_o_F_1_ activity that utilizes *pmf* for synthesizing ATP.

Although the molecular mechanism underlying its catalytic reactions of ATP synthesis/hydrolysis has been extensively studied over the past decades, the regulation mechanisms of F_o_F_1_ exhibit variation among species/organelles and remain unclear. To date, two inhibitory mechanisms, MgADP-induced inhibition (MgADP inhibition), which is conferred by the occupation of the catalytic site of the β subunit by MgADP, and ε-inhibition, an intrinsic inhibitory mechanism, have been shown to be conserved among bacterial F_o_F_1_s and are considered to be important for avoiding the futile ATP hydrolysis reaction and ensuring efficient ATP synthesis (5). In addition, in chloroplasts, it was previously demonstrated that ATP hydrolysis activity is regulated by intramolecular disulfide bond formation/dissociation in the γ subunit (6, 7, 8). However, in cyanobacteria, which are the phylogenetic ancestors of chloroplasts (9), this regulation does not occur, as the cyanobacterial γ subunit is devoid of the Cys residues, which are responsible for this redox regulation (10). Furthermore, the cyanobacterial regulatory mechanism for ATP synthesis reaction is still obscure.

F_o_F_1_ has a dual rotary-motor architecture, which couples the catalytic ATP synthesis/hydrolysis reactions with the electrochemical potential of H^+^ across the biological membrane (11, 12, 13, 14). F_o_F_1_ consists of a hydrophilic rotary motor, F_1_, and an intramembrane rotary motor, F_o_ (Fig. S1). F_1_ has a subunit stoichiometry of α_3_β_3_γ_1_δ_1_ε_1_, and the rotor shaft moiety composed of the γε subunits rotates inside of the stator subunits, α_3_β_3_, by the energy of ATP hydrolysis. Catalytic sites that are necessary for the ATP synthesis/hydrolysis reactions are present in the β subunit. The membrane-embedded F_o_ consists of three types of subunits with the stoichiometry of *a*_1_*b*_2_*c*_n_, where the rotor-ring composed of multimeric *c* subunits (*c*-ring) rotates relative to a stator part, *ab*_2_. The number of the *c* subunits in the ring varies among species, from 8 to 15 (15, 16, 17, 18, 19, 20, 21, 22). The γ subunit, a part of the rotor shaft of F_1_ motor, comprises N- and C-terminal helical domains, as well as a protruding globular Rossmann fold domain located between these two helical parts (Fig. 1*A*). The N- and C-terminal helical domains assemble into an antiparallel coiled-coil stalk, which is almost embedded in the central cavity of the catalytic headpiece, α_3_β_3_, as a rotor shaft. Moreover, the cyanobacterial and chloroplast γ subunits possess an inserted sequence of 30 or 39 amino acid residues within the Rossmann fold (Fig. 1, *A* and *B*) (10).

**Figure 1:**
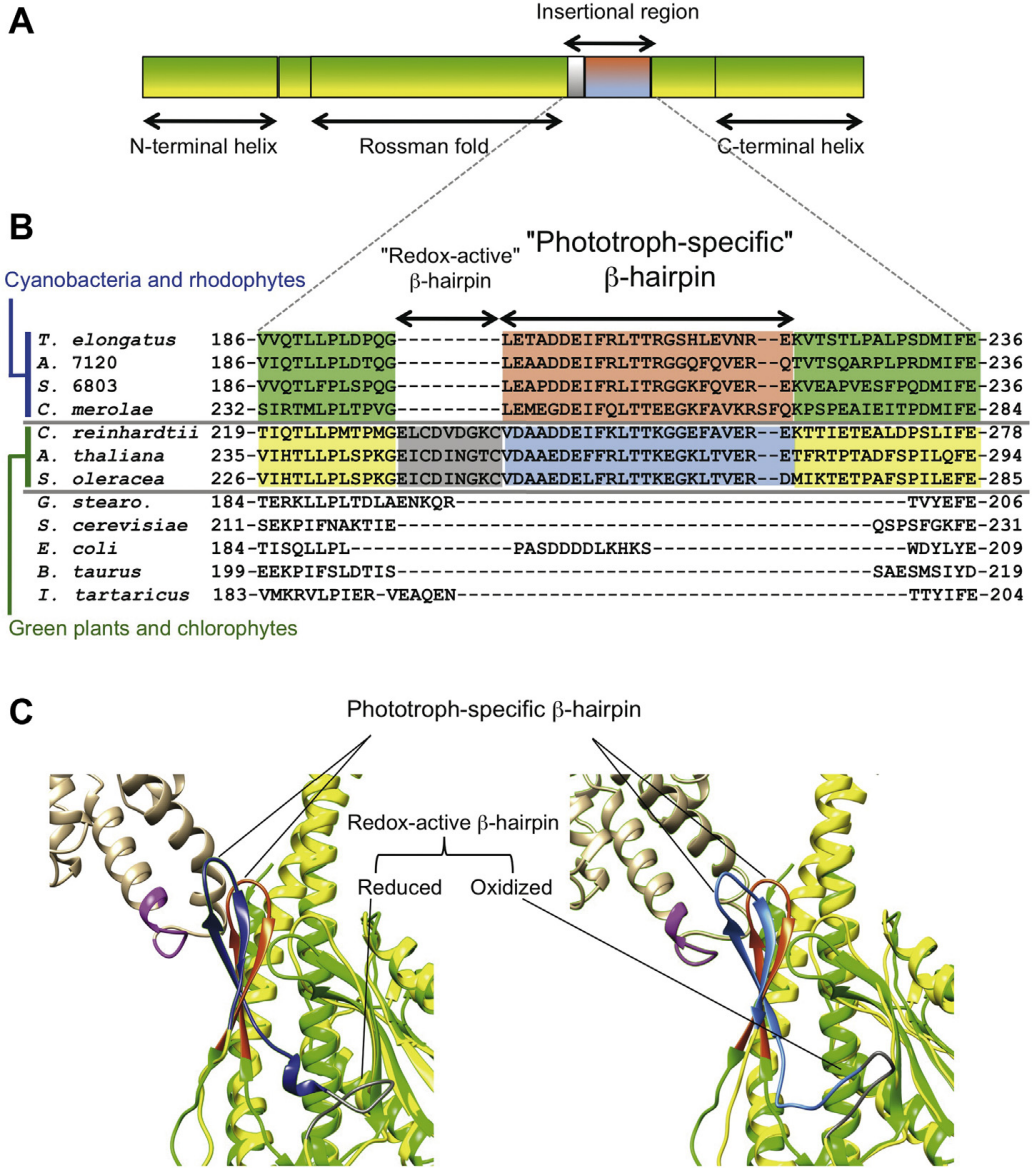
**Comparison of the γ subunits from various organisms.***A*, the domain architecture of the γ subunit from the phototrophs. They possess a unique insertion sequence within the Rossmann fold domain, between the N-terminal and C-terminal α helices. *B*, a partial alignment of amino acid sequences around the insertion region. The alignment was generated using the Clustal W multiple alignment tool. Cyanobacteria and rhodophytes have the phototroph-specific insertion, which forms a β-hairpin structure. Green plants and chlorophytes have an additional sequence, which forms the redox-active β-hairpin structure. *T. elongatus*, *Thermosynechococcus elongatus* BP-1; *A.* 7120, *Anabaena* sp. PCC 7120; *S.* 6803, *Synechocystis* sp. PCC 6803; *C. merolae*, *Cyanidioschyzon merolae*; *C. reinhardtii*, *Chlamydomonas reinhardtii*; *A. thaliana*, *Arabidopsis thaliana*; *S. oleracea*, *Spinacia oleracea*; *G. stearo*, *Geobacillus stearothermophilus* (formerly called *Thermophilic bacillus* PS3); *S. cerevisiae*, *Saccharomyces cerevisiae*; *E. coli*, *Escherichia coli*; *B. taurus*, *Bos taurus*; *I. tartaricus*, *Ilyobacter tartaricus*. *C*, schematic representations of the β-hairpin structures from cyanobacteria and chloroplasts. The γ subunit from *T. elongatus* was superimposed on that from *S. oleracea*; reduced (*left*) and oxidized (*right*) forms are shown. The *green* and *orange-red colors* indicate the γ subunit and the β-hairpin structure from *T. elongatus* (Protein Data Bank ID: 5ZWL); the *yellow* and *beige colors* indicate the γ subunit and the β subunit from *S. oleracea* (*left*, Protein Data Bank ID: 6VON; and *right*, 6VOH); the *blue*, *sky-blue*, and *gray colors* indicate the reduced and oxidized forms of the phototroph-specific β-hairpin structure, and the redox-active β-hairpin, from *S. oleracea*.

For a long time, the structure of this region remained unknown; however, since 2018, three papers have been published on this subject; two of them pertain to the structures of F_o_F_1_ from the chloroplasts of *Spinacea oleracea* and the other describes the structure of the γ–ε subcomplex from a thermophilic cyanobacterium, *Thermosynechococcus elongatus* BP-1 (*T. elongatus*) (23, 24, 25). It was revealed that this region forms a unique β-hairpin structure that extends along the central coiled-coil stalk and interacts with the “DELSEED” loop of the β subunit (Fig. 1*C*). This negatively charged loop is highly conserved among F_o_F_1_s, and its conformation is important for torque transmission from the catalytic site to the γ subunit upon ATP binding, thus affecting ATP hydrolysis activity (26, 27, 28). Only the chloroplasts from green plants and algae have an additional nine amino acids containing the above-mentioned redox-active Cys residues at the N terminus of the insertion region (Fig. 1*B*). It was shown that the ATP hydrolysis activity of the reduced form is significantly higher than that of the oxidized form (12, 29). In 2018, Hahn *et al.* (23) reported that this sequence forms an additional small β-hairpin structure. Moreover, Yang *et al.* (25) recently published the structures of both oxidized/reduced F_o_F_1_ from spinach chloroplasts. In view of the molecular structure, the interactions between the DELSEED-loop of the β subunit and the β-hairpin structure of the γ subunit did not differ significantly between the oxidized and reduced states (25). It is possible that, in cyanobacteria, which are devoid of this small redox-active β-hairpin, the interaction between the DELSEED loop of the β subunit and the β-hairpin structure of the γ subunit differs from that of chloroplasts. As shown in Figure 1*C*, the tilting angle of the cyanobacterial β-hairpin appears to be slightly different from that of both the oxidized and reduced forms of the spinach γ subunit. As the only structure of the cyanobacterial γ subunit that has been published to date is the γ–ε subcomplex (24), its interaction with the DELSEED loop is not fully understood.

In our previous reports, using recombinant α_3_β_3_γ derived from *T. elongatus*, we demonstrated that the phototroph-specific insertion region plays an important role in *MgADP inhibition* (30). The whole-β-hairpin-truncated α_3_β_3_γ (α_3_β_3_γ^Δ198–222^) exhibited higher ATP hydrolysis activity than the wildtype form. We also demonstrated that the two-amino-acid truncations at the turn of the β-hairpin structure (α_3_β_3_γ^Δ212–213^) resulted in a significant increase in ATP hydrolysis activity *via* the cancelation of MgADP inhibition. Truncation of an even greater number of amino acids did not raise the activity further (α_3_β_3_γ^Δ211–214^, α_3_β_3_γ^Δ210–215^, α_3_β_3_γ^Δ209–216^, and α_3_β_3_γ^Δ205–220^) (24). These results strongly suggested that the interaction between the DELSEED loop of the β subunit and the two amino acids located at the turn of the β-hairpin structure is sufficient to induce MgADP inhibition upon ATP hydrolysis. This model was supported by our studies using recombinant α_3_β_3_γ derived from *T. elongatus* with nick insertion into the proximal region of the β-hairpin structure (between V^222^ and T^223^) and cross-linking experiments using disulfide bond formation between the central stalk and the β-hairpin structure within the γ subunit (31, 32).

Those findings prompted us to investigate the correlation between the β-hairpin structure and ATP synthesis and H^+^-translocation in cyanobacterial F_o_F_1_. To date, the role of this structure in ATP synthesis and H^+^-translocation has not been elucidated, partly because those measurements require the preparation of the reproducible quality of proteoliposomes (PLs) and quantitative application of Δ*μ*H^+^ across the membrane. Here, we set up a one-step mild purification method of cyanobacterial F_o_F_1_ from *Synechocystis* sp. PCC 6803 (*S.* 6803). *S.* 6803 is a mesophilic cyanobacterium that allows easy genetic engineering, as this strain is capable of natural transformation with high efficiency in double homologous recombination and its genome harbors sufficient homology to *T. elongatus*: their amino acid sequences of the γ subunit share 73% homology (Fig. 2*A*). We analyzed the ATP hydrolysis/synthesis and H^+^-translocating activities of the wildtype and β-hairpin-truncated F_o_F_1_ mutants. Based on the detailed biochemical analyses of these phenomena using phototroph-derived F_o_F_1_, we here describe the role of the conserved β-hairpin structure in ATP synthesis. Our results demonstrated that the phototroph-specific β-hairpin structure of the γ subunit of F_o_F_1_ critically contributes to its ATP synthesis activity, in addition to suppressing ATP hydrolysis. We also performed physiological analyses using *S.* 6803 and found that the intracellular ATP content was significantly decreased in the β-hairpin-truncated mutants. However, under our experimental conditions, there was no noticeable effect other than the decrease in the amount of ATP.

**Figure 2:**
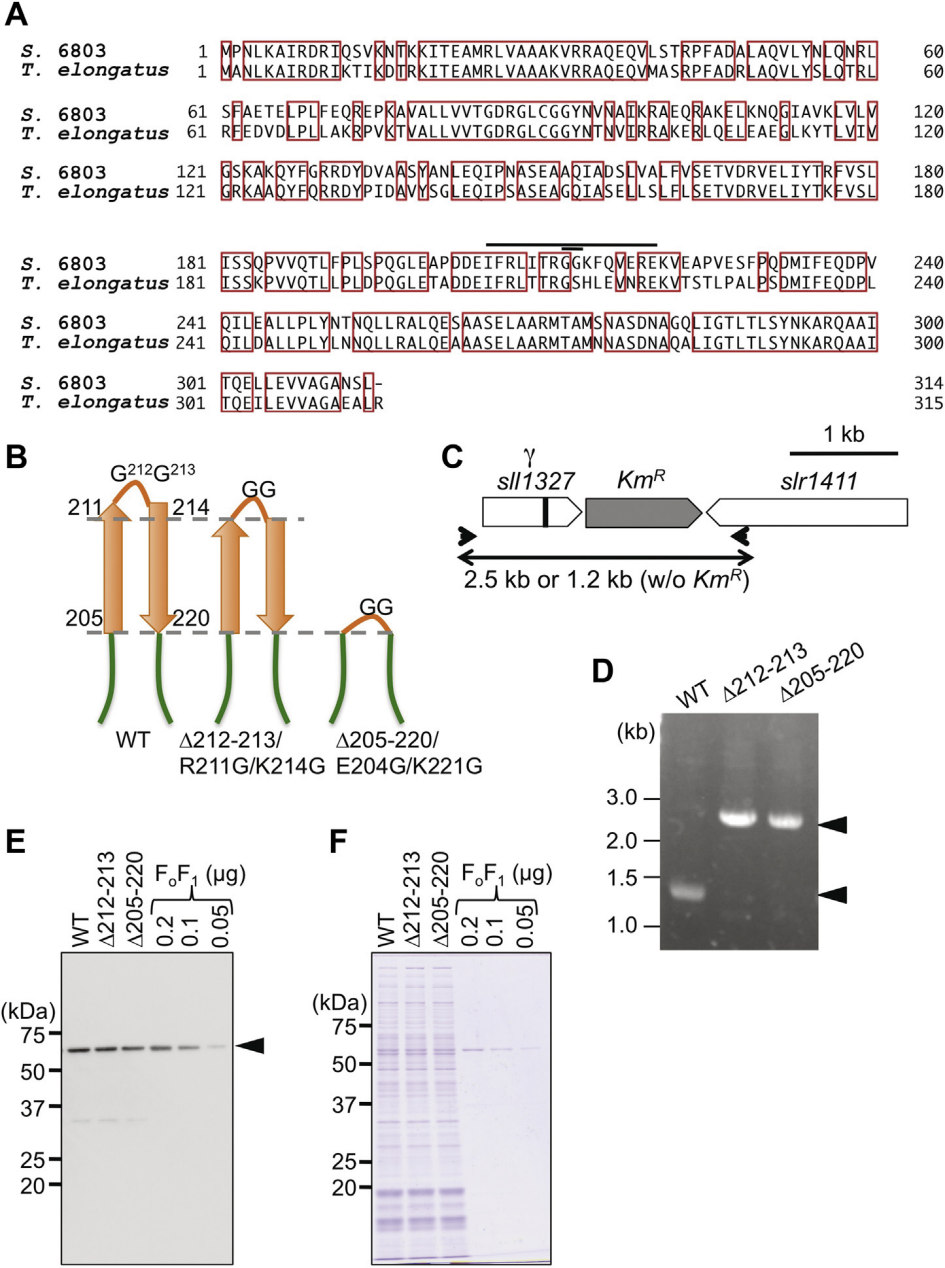
**Construction of the β-hairpin-truncated mutants of *S*. 6803.***A*, amino acid sequences of the γ subunit from *S.* 6803 and *T. elongatus*. Conserved residues are highlighted in *red boxes*. The truncated regions, *i.e.*, 212–213 and 205–220, are indicated by *black lines*. This region was previously shown to form a β-hairpin structure, which is specific to cyanobacteria and chloroplasts (*B*). Two amino acids located at the turn of the β-hairpin structure were truncated, along with the R211G and K214G substitutions, to maintain the β-hairpin structure, in Δ212–213/R211G/K214G (Δ212–213), whereas the whole β-hairpin structure was truncated in Δ205–220/E204G/K221G (Δ205–220). *C*, schematic diagram of the gene arrangement expected in the mutants. The position of the truncated region in *sll1327*, which encodes the γ subunit, is indicated by a *black box*. *D*, PCR analysis of the genomic DNA of transformants verified complete segregation of the mutated gene. The primers used to amplify the sequence of interest are indicated in *C* by *black arrowheads*. *E* and *F*, immunoblot analysis using β subunit antibodies (*E*) and SDS-PAGE (*F*) in *S.* 6803 cells from WT, Δ212–213, and Δ205–220 strains. Cell crude extracts of 80 ng of chlorophyll were loaded per lane. A total of 0.05 to 0.2 μg of the wildtype F_o_F_1_ was used as a control.

## Results

### Purification of F_o_F_1_ ATP synthase and construction of the β-hairpin-truncated mutants of the γ subunit

**Figure 3:**
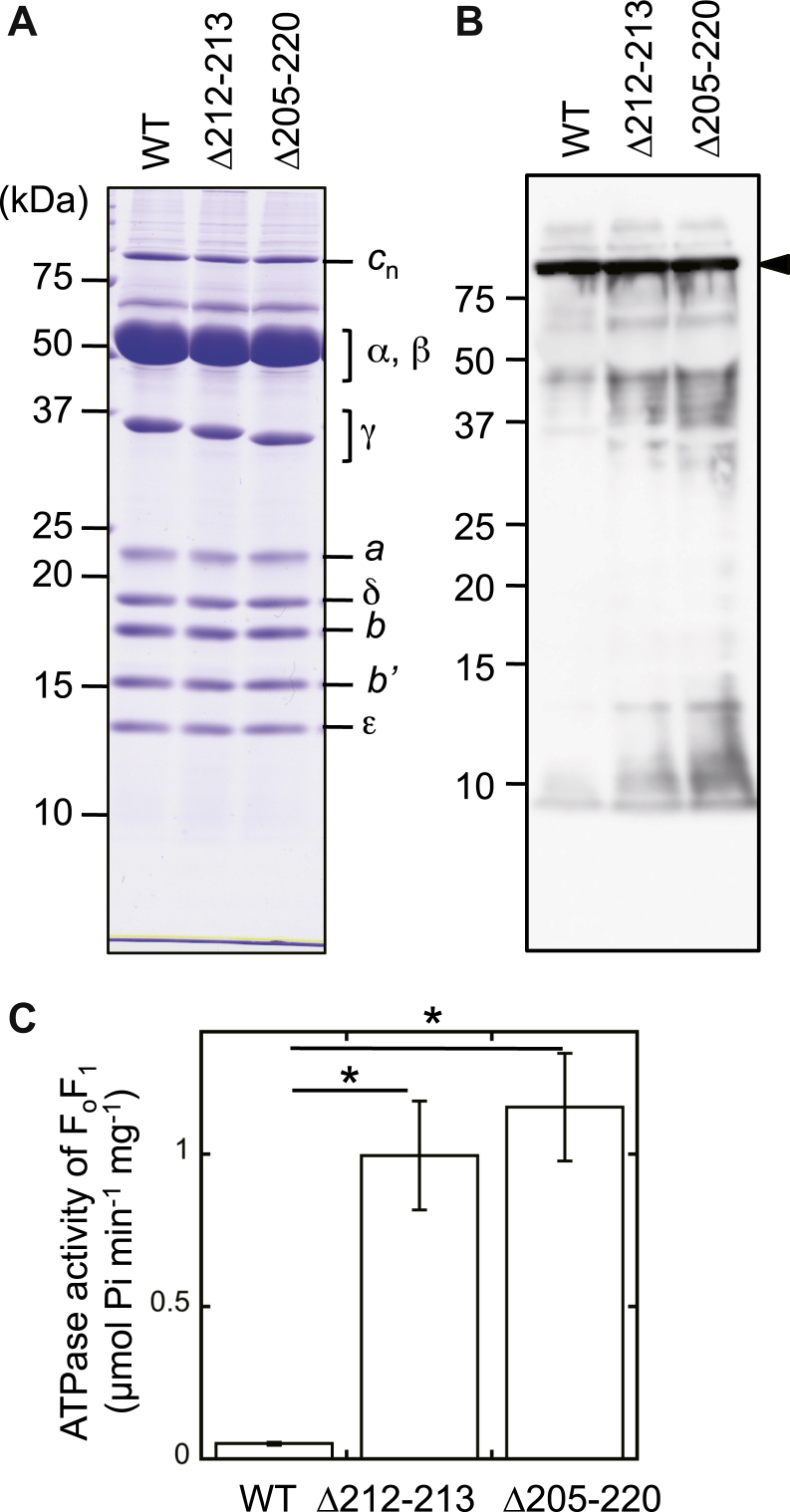
**Purification of F**_**o**_**F**_**1**_**ATP synthase and measurements of the ATP hydrolysis activity of reconstituted proteoliposomes.***A*, purity of the F_o_F_1_ complexes. The complex was purified by membrane solubilization with lauryl maltose neopentyl glycol, a nonionic detergent, and subsequent nickel affinity chromatography. Then, 10 μg of proteins was loaded per lane. *B*, western blot detection of the *c*-ring. The proteins electrophoresed on 16% polyacrylamide gels were transferred onto a PVDF membrane, followed by immunoblotting with specific anti-*c*-subunit antibodies; chemiluminescence was measured. *C*, ATP hydrolysis activity of proteoliposomes was measured using an ATP-regenerating system. The assay was conducted at 30 °C. The activities were determined from the slope obtained in the steady state. The results of four independent experiments were averaged (mean ± SD). The *asterisks* indicate statistical significance (*p* < 0.005, Welch’s *t* test).

To investigate the role of the β-hairpin on ATP synthesis function, we purified F_o_F_1_ preparations from *S*. 6803, as follows. First, the His_10_-tag sequence was genetically fused to the N terminus of the β subunit in *S.* 6803 (Fig. S2*A*). The resulting *S.* 6803 was termed WT strain in the present study. Then, thylakoid membranes were prepared from the WT strain and solubilized with lauryl maltose neopentyl glycol (LMNG). The F_o_F_1_ preparation was purified from the solubilized fraction by Ni-affinity chromatography. The resulting F_o_F_1_ preparation was subjected to SDS-PAGE (Fig. 3*A*, lane WT). Nine types of bands were observed on the gel, which were assumed to correspond to the nine subunits of WT-F_o_F_1_. The band intensity of the eight bands other than that corresponding to the *c* subunit agreed with the expected subunit stoichiometry of *S.* 6803 F_o_F_1_, *i.e.*, α_3_β_3_γεδ*abb’c*_n_. The validity of the bands was confirmed by peptide mass fingerprinting after trypsin digestion, N-terminal sequencing, or immunoblot analyses (Table 1 and Fig. 3*B*). The SDS-PAGE analysis additionally identified a band corresponding to the *c* subunit above that of the α subunit on the gel (Fig. 3, *A* and *B*). As observed previously in the *c* subunit of *Propionigenium modestum* (33), the *c* subunits of *S.* 6803 formed a stable *c*-ring architecture that was resistant to the electrophoretic analysis, although the number of *c* subunits in the ring remained obscure (which was previously reported as 14 in the literature) (21). Furthermore, N-terminal sequencing of the *a* subunit pinpointed the location of the proteolytic cleavage position between Ala^42^ and Ala^43^. Based on the genomic sequence of the *a* subunit in the KEGG database (https://www.genome.jp/kegg/; accession number, sll1322), an N-terminal 42-residue segment is suggested to be processed. However, a multiple sequence alignment showed that an N-terminal region composed of 28 residues in *S.* 6803 was not present in those of other cyanobacteria. This result suggests that the translation of the *a* subunit starts at a second methionine, Met^29^, of the open reading frame provided in the database (Fig. S3). If so, the 14 N-terminal residues are the correct segment, which is processed by proteolytic digestion in *S.* 6803. Because the N terminus of the *a* subunit is located on the lumenal side of thylakoid membranes (23), the N-terminal 14-residue segment is expected to act as a signal peptide for transport across the membrane. However, further analyses are necessary to confirm this function.

**Table 1:** Subunits of the F_o_F_1_ ATP synthase of *S.* 6803 identified by peptide mass fingerprint analysis and N-terminal sequencing analyses

Subunit	ID	Theoretical mass	MALDI-TOF MS		N-terminal sequencing^a^
			Score^b^	Coverage (%)	
α	sll1326	54,000	97	24	VXIXPDEISS
β	slr1329	53,100^c^	188	44	Not detected
γ	sll1327	34,600	124	39	PNLXAIXD
*a*	sll1322	30,700	Not detected		ALEVGQ
δ	sll1325	20,000	98	70	MXGSLY
*b*	sll1324	19,800	104	29	Not detected
*b'*	sll1323	16,200	100	30	Not detected
ε	slr1330	14,600	56	39	TLTVRVIT
*c*	ssl2615	8000	Not detected		Not detected

a Undetermined amino acids are indicated with an “X.”

b Score obtained by Mascot (http://www.matrixscience.com).

c Theoretical mass, including the His-tag.

Next, we prepared two mutated strains of *S.* 6803, Δ212–213 and Δ205–220, *via* the genome manipulation of WT-*S.* 6803 (Fig. 2, *B* and *C*). The complete segregation and replacement of endogenous *sll1327*, which encodes the γ subunit, with the mutated gene were validated by PCR analysis (Fig. 2*D*) and DNA sequence analysis. The mutants carried the genetic deletion of Δ212–213 or Δ205–220 of the γ subunit, respectively. Therefore, the two amino acids located at the tip of the turn region or the entire β-hairpin structure were not present in the two mutants (Fig. 2*B*). We then purified the mutant F_o_F_1_ using the same procedure as that used for the WT strain. No significant differences were observed in subunit stoichiometry (Fig. 3, *A* and *B*) or expression level, which was assessed based on immunoblotting using β subunit–specific antibodies (Fig. 2, *E* and *F*), between the WT and the mutant strains.

The extent of MgADP inhibition was assessed by the ratio of activation of ATP hydrolysis by lauryl dimethylamine oxide (LDAO). LDAO is a nonionic detergent and considered to be effective in the release of F_1_ from the MgADP-inhibited state (34). For this purpose, the F_1_-enriched (“crude F_1_”) fractions were obtained by chloroform extraction (Fig. S4*A*). The results indicated that the Δ212–213 and Δ205–220 mutations led to a significant increase of ATP hydrolysis activity (Fig. S4*B*), and LDAO activation ratios were significantly lower than those from WT (Fig. S4*C*), which is consistent with the results from *T. elongates* (24). These results indicate that ATP hydrolysis was inhibited by MgADP in WT.

### Preparation of PLs and measurement of ATP hydrolysis/synthesis activities

**Figure 4:**
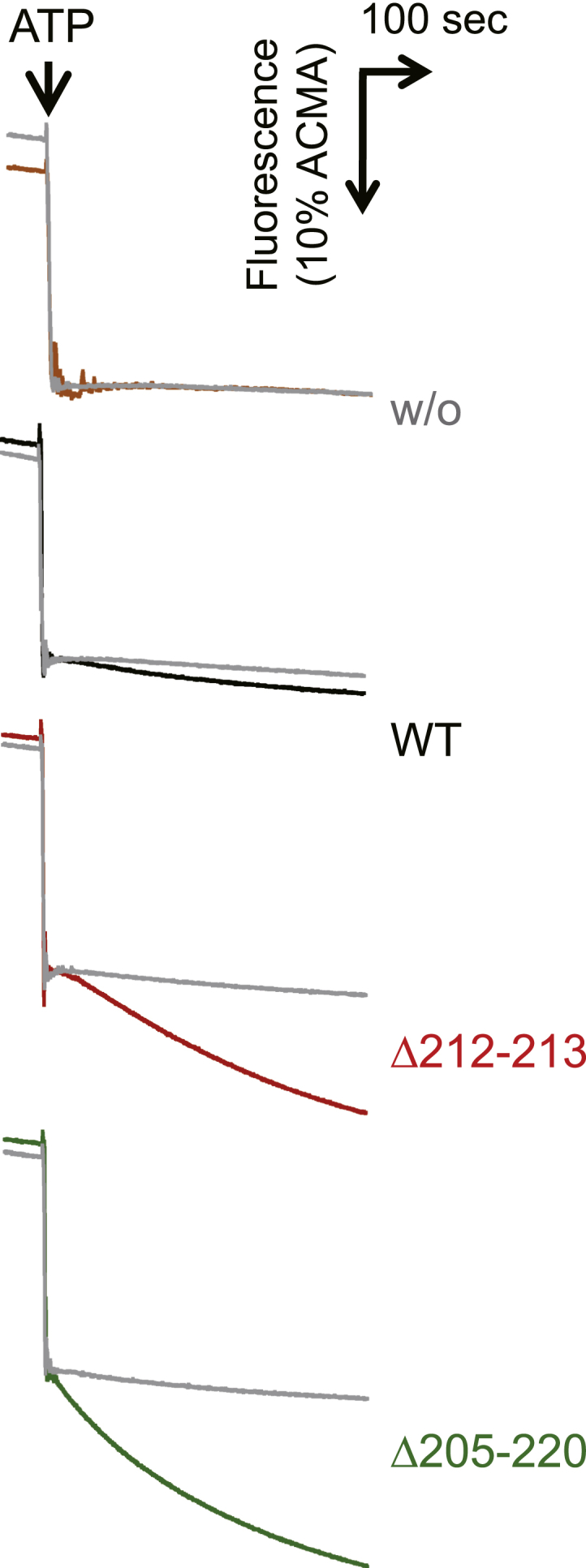
**Measurements of the proton-translocating activity of proteoliposomes.** The reaction was initiated by the addition of 2 mM ATP to proteoliposomes containing F_o_F_1_ with (*gray*) or without 1 μg ml^−1^ FCCP at 30 °C. The inside acidification was monitored with fluorescence quenching of ACMA (excitation at 410 nm, emission at 480 nm). Liposomes were used as the negative control (w/o).

The obtained F_o_F_1_ preparations were, respectively, reconstituted into soybean liposomes to obtain F_o_F_1_ PLs. As observed in the analyses of α_3_β_3_γ derived from *T. elongatus* (24), ATP hydrolysis activities of the mutant PLs were higher than those of the WT PLs (Fig. 3*C*). The activity was roughly 20- and 23-fold in Δ212–213 (1.0 ± 0.2 μmol Pi min^−1^ mg^−1^) and Δ205–220 (1.2 ± 0.2 μmol Pi min^−1^ mg^−1^) compared with the WT PLs (0.051 ± 0.005 μmol Pi min^−1^ mg^−1^) (Fig. 3*C*). The degree of the enhancement was similar in the two mutants. Subsequently, H^+^-translocating activity of the PLs was analyzed using a ΔpH indicator, ACMA (Fig. 4). The addition of ATP drove its hydrolysis, followed by coupled proton translocation into PLs, which was observed as the fluorescence quenching of ACMA. The Δ212–213 and Δ205–220 PLs showed a higher proton-translocating activity than WT PLs, the activity of which was diminished in the presence of the H^+^-ionophore FCCP. However, the increases of the activity were relatively moderate when compared with those of the ATP hydrolysis analysis: slopes of the quenching were 0.0066 ± 0.0015, 0.025 ± 0.0094, and 0.049 ± 0.017 IU s^−1^ for WT, Δ212–213, and Δ205–220, respectively (average ± SD, n = 3).

**Figure 5:**
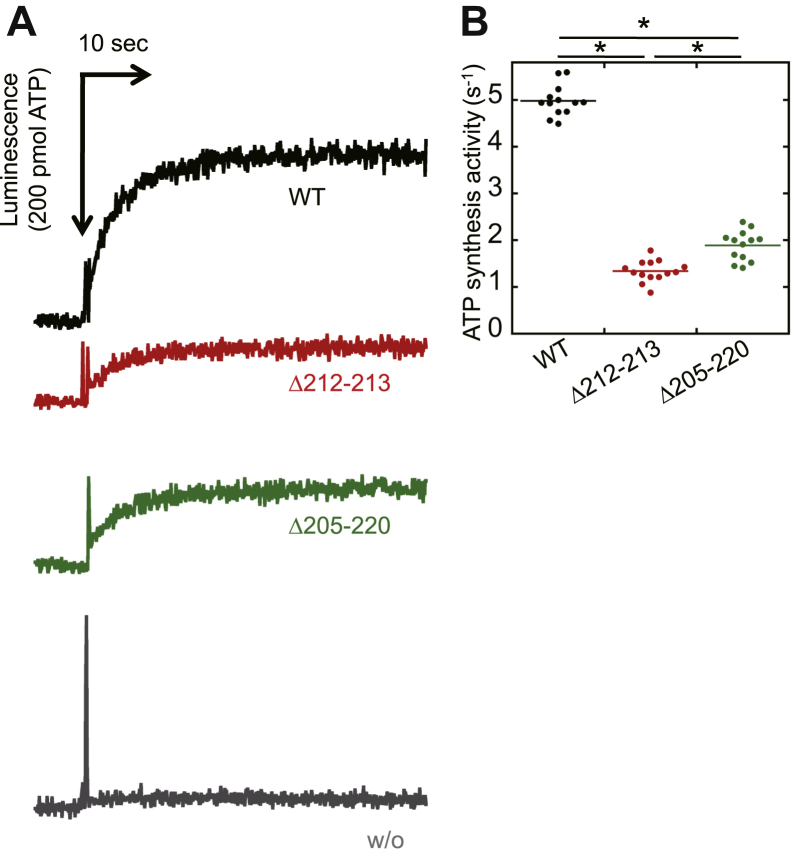
**Measurements of the ATP synthesis activity of proteoliposomes.** The synthesis reaction was initiated by the injection of acidified proteoliposomes. The *vertical axis* indicates luminescence from luciferin. The intensity was converted to the amount of ATP in the reaction mixture by calibration with the addition of ATP after each measurement (*A*). The initial velocity of synthesis (s^−1^) was calculated from the exponential fit from 0 to 45 s after the injection. Liposomes were used as the negative control (w/o). The results of 13 (WT and Δ205–220) or 14 (Δ212–213) independent experiments are shown as a dot plot (*B*). The *horizontal lines* indicate the average. The *asterisks* indicate statistical significance (*p* < 0.001, Welch’s *t* test).

ATP synthesis activities were then analyzed using the acid–base transition method with valinomycin-induced diffusion potential of K^+^ (Fig. 5*A*) (35, 36) as follows: first, PLs were incubated with an acidic buffer (pH 5.6) to acidify the inside of the PLs. The acidified PLs were rapidly injected into the assay mixture, which included a basic buffer (pH 8.8), K^+^, ADP, Pi, and luciferin/luciferase. The increase in luminescence intensity was monitored in real time at 30 °C using a luminometer (see the Experimental procedures section for details). The injection of PLs into the assay mixture resulted in the formation of ΔpH between the inside and outside of PLs. Simultaneously, the injection induced the diffusion of K^+^ from the outside to inside of PLs with the assistance of valinomycin, resulting in the formation of an inside-positive Δ*Ψ* across the lipid bilayer of PLs. These two energies, termed ΔpH and Δ*Ψ*, are driving forces of ATP synthesis by F_o_F_1_ (37). The amounts of ATP synthesized in the reaction mixture were calibrated by the addition of 50 pmol ATP three times at the end of the measurements. The activity was given as the initial rate of synthesis, which was obtained by fitting to the following equation:

$$y\;=\;y_{\text{0}}\;+\;a\text{ × }\left[1-\text{exp}\left\{-b\text{ × }\left(x-x_{\text{0}}\right)\right\}\right]$$

over 0 to 45 s after the addition of PLs. As a result, the ATP synthesis activity was 5.0 ± 0.3, 1.3 ± 0.2, and 1.9 ± 0.3 s^−1^ for WT, Δ212–213, and Δ205–220, respectively (average ± SD, Fig. 5*B*). We further confirmed that those activities were abolished by the addition of the uncoupler nigericin (Fig. S5).

**Figure 6:**
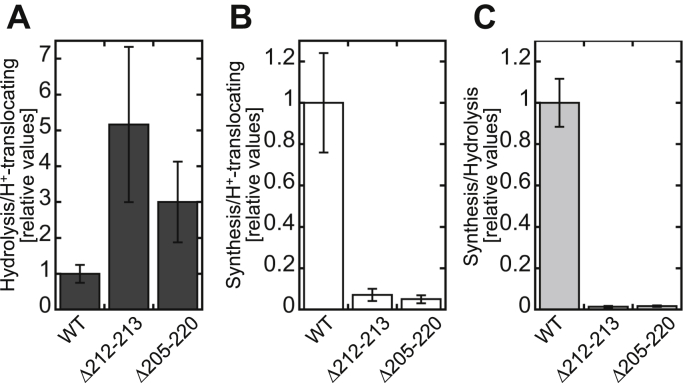
**Comparative analyses of ATP hydrolysis/synthesis and proton-translocating activities.***A*, the relative ratio of ATP hydrolysis to H^+^-translocating activities, compared with WT. The ATP hydrolysis activities shown in Figure 3*C* were converted to s^−1^ (0.48 ± 0.05, 9.4 ± 1.7, and 11 ± 1.7 for WT, Δ212–213, and Δ205–220, respectively). The relative H^+^-translocating activities were calculated from the slope obtained in the steady state (Fig. 4, 0.0066 ± 0.0015, 0.025 ± 0.009, and 0.049 ± 0.017 IU s^−1^ for WT, Δ212–213, and Δ205–220, respectively). *B* and *C*, the relative ratio of ATP synthesis to H^+^-translocating activities and the relative ratio of ATP synthesis to ATP hydrolysis activities, compared with WT. The ATP synthesis activities shown in Figure 5*B* were used for calculation (5.0 ± 0.3, 1.3 ± 0.2, and 1.9 ± 0.3 s^−1^ for WT, Δ212–213, and Δ205–220, respectively).

Based on the results reported above, we demonstrated that, in the two β-hairpin-truncated mutants, *i.e.*, Δ212–213 and Δ205–220, the ATP hydrolysis activity was increased, and the ATP synthesis activity was decreased, compared with the WT strain. This raised the possibility that the β-hairpin truncation led to the functional uncoupling between F_o_ and F_1_ motors, *i.e.*, the energy (torque) obtained from *pmf* at F_o_ cannot be sufficiently transferred to F_1_’s catalytic site to synthesize ATP, because of the structural instability resulting from the truncation. However, as contradicted to this interpretation, those mutants showed significant increases in ATPase-driven H^+^-translocating activity by the mutation (3.8-fold in Δ212–213 and 7.5-fold in Δ205–220) (Fig. 4). This suggests that the regulative effect, rather than the simple uncoupling effect, confers the above-mentioned feature to those mutants. Therefore, we evaluated the rate of uncoupling by comparing the ATP hydrolysis/synthesis and proton-translocation activities shown in Figures 3*C*, 4 and 5*B*. Figure 6*A* indicates the relative ratio of ATP hydrolysis to H^+^-translocating activities. The relative ratios to the WT obtained for Δ212–213 and Δ205–220 were 5.2 ± 2.2 and 3.0 ± 1.1. This suggests that, in those mutants, F_o_F_1_ was partially uncoupled to different extents and that the uncoupling effect was stronger in Δ212–213 than it was in Δ205–220. Figure 6, *B* and *C* indicates the relative ratios of ATP synthesis to H^+^-translocating activities and the relative ratios of ATP synthesis to ATP hydrolysis activities, respectively, both of which were remarkably decreased in Δ212–213 and Δ205–220 compared with the WT; this shows that the preference of F_o_F_1_ between ATP synthesis and ATP hydrolysis significantly shifted from ATP synthesis to ATP hydrolysis in the two mutants. These calculations led us to conclude that the β-hairpin structure is a regulatory module that facilitates the achievement of efficient ATP synthesis by F_o_F_1_.

### The physiological effect of the β-hairpin truncation

**Figure 7:**
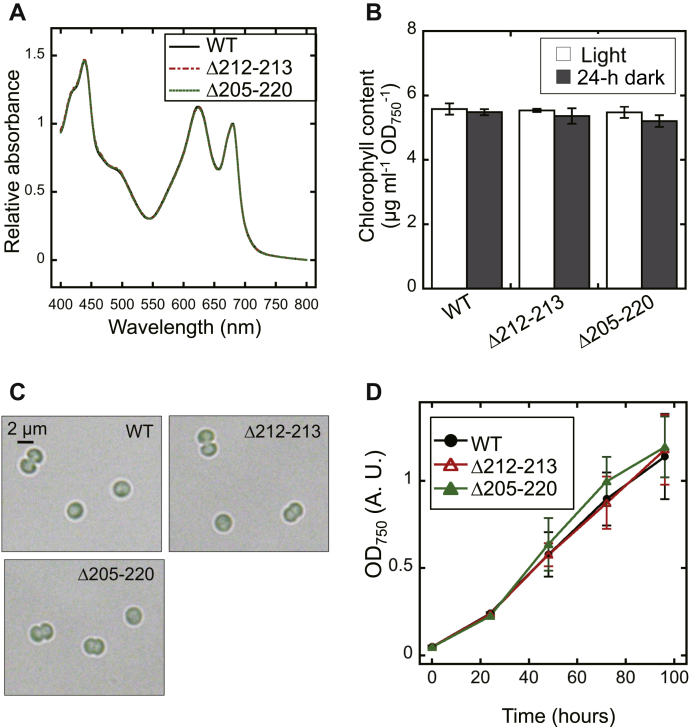
**Physiological analyses of the *S.* 6803 mutants, Δ212–213 and Δ205–220.***A*, absorption spectra of WT, Δ212–213, and Δ205–220 strains grown under continuous light conditions (40 μmol photons m^−2^ s^−1^). Cells were precipitated and resuspended in BG11 medium (OD_750_ = 1.0), followed by measurements using a spectrophotometer equipped with an integrating sphere. Each spectrum was normalized at 680 nm. *B*, chlorophyll content of cells grown under continuous light conditions or after a 24-h dark treatment, measured by methanol extraction. None of these results were significantly different from each other (Tukey–Kramer multiple comparison tests; *p* > 0.05). *C*, optical microscopical analysis of cells grown under continuous light conditions. *D*, growth curves obtained under continuous light conditions (40 μmol photons m^−2^ s^−1^) with ambient CO_2_ at 30 °C, as assessed by measuring optical density at 750 nm (OD_750_). Data are the mean ± SD from 3 to 5 independent experiments (*B* and *D*).

Next, we investigated the physiological effect of β-hairpin truncation in cyanobacterial cells. The absorption spectra revealed that pigmentation was not affected in cyanobacterial mutant cells (Fig. 7*A*), as there was no difference in chlorophyll concentration between WT and the mutant cells (Fig. 7*B*). We also confirmed that the size of those cells was almost identical (1.89 ± 0.15, 1.96 ± 0.13, and 1.89 ± 0.12 μm/cell for WT, Δ212–213, and Δ205–220, n = 30; Fig. 7*C*). These observations permitted us to estimate intracellular compounds *via* normalization either to chlorophyll content or cell density, which was reflected in the optical density at 750 nm (OD_750_).

**Figure 8:**
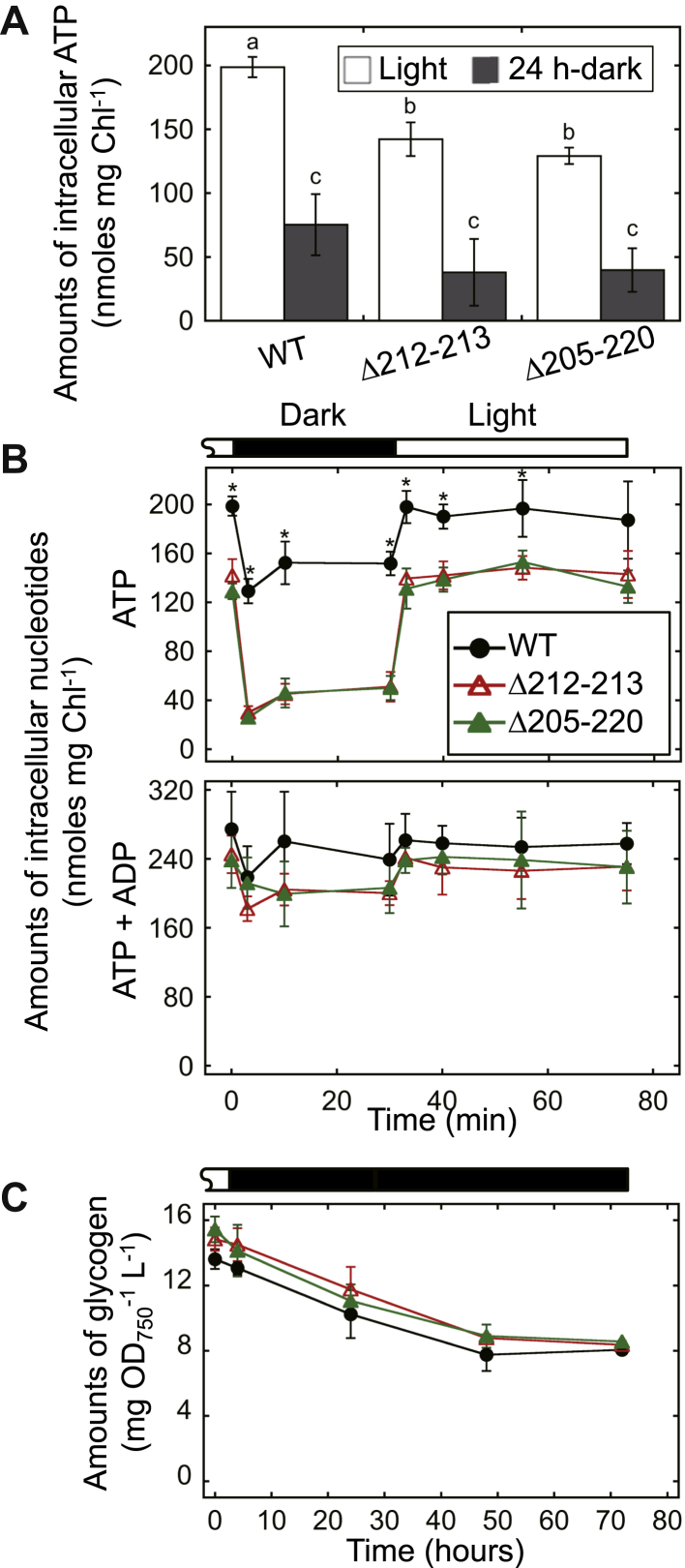
**Measurements of intracellular ATP (+ADP) and glycogen content of the *S.* 6803 mutants, Δ212–213 and Δ205–220.***A*, steady-state intracellular ATP content normalized to chlorophyll content (Chl). Cells were grown under continuous illumination (Light), or followed by dark treatments for 24 h (24-h dark) and fixed with 2% (w/v) perchloric acid. After neutralization, ATP in the supernatant was quantified using a luciferin–luciferase assay. *Different letters* (a, b, and c) indicate significant differences between them (*p* < 0.05, Tukey–Kramer multiple comparison tests). *B*, short-term kinetics of intracellular ATP or ATP + ADP levels during a light–dark–light transition. Cells grown under continuous illumination were treated with dark incubation for 30 min. At the indicated times, cells were fixed with 2% (w/v) perchloric acid, followed by neutralization and quantification. For quantification of ATP + ADP, before the luciferin–luciferase assay, neutralized aliquots were incubated for 3 h at 25 °C in the presence of pyruvate kinase. The *asterisks* indicate statistical significance (*p* < 0.05, Welch’s *t* test). *C*, the amount of glycogen in dark-incubated cells. At the indicated times, cells were collected by centrifugation, followed by incubation at 100 °C for 120 min in 3.5% sulfuric acid. The resultant glucose levels were determined by LabAssay Glucose (Wako) according to the manufacturer’s instructions. Data are the mean ± SD from 3 to 5 independent experiments.

As shown in Figure 7*D*, there was no significant difference in the growth rates between the WT and the mutant cells under continuous light conditions. Sunamura *et al.* previously reported that the γ_Δ198–222_ mutant of *S.* 6803, in which the whole γ-insertion region was deleted, exhibited a reduction of the intracellular ATP content and that the growth of γ_Δ198–222_ was not significantly different from that of the wildtype cells under continuous light conditions and light/dark (8/16 h) conditions (30). The present results shown in Figure 7*D* were analogous to the previous results as expected. Here, we investigated the intracellular ATP content of the mutants, *i.e.*, Δ212–213 and Δ205–220, and further explored the physiological effect of the decrease in ATP level. Figure 8*A* indicates the steady-state level of intracellular ATP content. Under continuous light conditions, the ATP levels were decreased to 72% in Δ212–213 and 65% in Δ205–220 compared with the WT (Fig. 8*A*, white bars). The differences in ATP level relative to the WT increased after dark treatment for 24 h, to 50% and 53%, respectively (Fig. 8*A*, gray bars). We then investigated the short-term kinetics of both ATP and ADP content during a light-to-dark transition (Fig. 8*B*). As reported in the literature, in the WT cells, the ATP content was decreased within a few minutes after the transfer from the light to the dark. During the dark period, in cyanobacterial cells, the ATP level was gradually increased by oxidative phosphorylation (38). Similar to previous reports (30), the ATP content was decreased in Δ212–213 and Δ205–220 compared with the WT. The two analyses depicted in Figure 8, *A* and *B* showed that the ATP level was not significantly different between the two mutants under light or dark conditions. The light-to-dark transition analysis yielded the following remarkable finding: the degree of the relative decrease in ATP level in the mutants was more significant than that observed in the WT. Although we analyzed the photosynthetic activity under the conditions where the ATP levels were significantly affected in the mutants (Fig. S6), there were no remarkable differences between WT and the mutants. Furthermore, we found that the total amount of ATP + ADP was comparatively stable over the experimental period (Fig. 8*B*), indicating that the dynamics of ATP level reported above mainly reflect the conversion of ADP/ATP. Under continuous light conditions, the ratio of ATP to ATP + ADP was calculated as 73%, 58%, and 57% in the WT, Δ212–213, and Δ205–220 strains, respectively. After a 24-h incubation in the dark, this ratio was decreased to 40%, 23%, and 30%, respectively. These results prompted us to investigate the effects of this phenomenon on the bioenergetic metabolism. Figure 8*C* depicts the intracellular glycogen content under continuous light and dark conditions. Glycogen levels were gradually reduced after transfer from the light to the dark, which resulted from respiration and autofermentation (39). Contrary to expectations, despite the significant decrease in ATP level, the results obtained for the mutants were not significantly different from those recorded for WT cells.

## Discussion

### The phototroph-specific β-hairpin structure of *S.* 6803 facilitates the synthesis and suppresses the hydrolysis of ATP

Here, we established a single-step method for the preparation of *S.* 6803 F_o_F_1_. There were no significant differences in the subunit composition and stoichiometry of the F_o_F_1_ preparation compared with those of the enzyme purified using the conventional method (40). The obtained F_o_F_1_ was reconstituted into soybean liposomes to analyze its functions in detail. The resulting F_o_F_1_ PLs exhibited sufficient activity for the comparative analyses of ATP synthesis/hydrolysis and proton translocation between the WT and the mutant strains. Targeted genome manipulation is relatively easy in *S.* 6803, whereas it is possible but not easy in some other cyanobacteria, green algae, and higher plants. In the present study, we investigated for the first time the role of the phototroph-specific β-hairpin region of F_o_F_1_ in the ATP synthesis/hydrolysis activity of the enzyme. Although we could not exclude the possibility that a certain amount of mutant F_o_F_1_ was uncoupled, especially in the Δ212–213 mutant, the accelerated H^+^-translocating activities of the mutants and data analyses allowed us to conclude that ATP synthesis was suppressed and ATP hydrolysis was accelerated in the β-hairpin-truncated mutants. Overall, the results presented here indicate that the β-hairpin region of the γ subunit of F_o_F_1_ from *S.* 6803 critically contributes to its ATP synthesis activity and suppresses ATP hydrolysis.

Because the synthesis and hydrolysis of ATP by F_o_F_1_ are thermodynamically reversible, it is not readily explainable why the β-hairpin structure oppositely affected those two reactions. In contrast, MgADP inhibition is a regulatory mechanism that achieves such contradictory functions. As mentioned above, this inhibitory mechanism results from the persistent occupation of the catalytic site by MgADP derived from ATP hydrolysis and is spontaneously canceled during ATP synthesis. This is consistent with our previous findings that MgADP inhibition was partly canceled in the β-hairpin-truncated mutants (24). Those findings led us to conclude that the phototroph-specific β-hairpin structure suppresses ATP hydrolysis by facilitating MgADP inhibition. This was possibly explained by the interaction between the DELSEED region of the β subunit and a part of the γ subunit, although structural studies are necessary to prove this hypothesis. It was recently reported that the ε subunit of cyanobacterial F_o_F_1_ has a different inhibitory mechanism compared with other organisms (41) and that the binding of the ε subunit caused a relative conformational change in the γ subunit (32). Consequently, the β-hairpin structure of the *S.* 6803 γ subunit, assisted by the ε subunit, might confer stiffness to the γ subunit and facilitate torque transmission during ATP synthesis.

### Regulation of the intracellular ATP level in cyanobacteria

As shown in Figure 8*A*, the intracellular ATP level was significantly decreased in both Δ212–213 and Δ205–220 compared with the WT cells. After a 24-h dark adaptation, the ATP levels in those mutants were decreased to 19% to 20% of the normal level (*i.e.*, WT, continuous illumination conditions). These results support our hypothesis that the β-hairpin structure plays a role in the inhibition of ATP hydrolysis and the acceleration of ATP synthesis. We further demonstrated that their cell shape/size, pigmentation, growth rate, and glycogen content were not significantly different from those of the WT cells (Figs. 7, *A*–*D* and 8*C*). This was very similar to the case of IF1, which is an inhibitory protein of mitochondrial ATP synthase and considered to play a role in preventing futile ATP hydrolysis (42). Contrary to previous expectations, it was reported that IF1-knockdown or knockout cells did not show any apparent phenotype, except after the exposure of cells to extreme conditions (*e.g.*, high concentrations of reactive oxygen species) (43, 44). In cyanobacteria, Forchhammer *et al.* reported that the intracellular ATP level was strictly regulated during the awaking phase from dormancy caused by nitrogen chlorosis (45, 46). Those authors argued that the dormant cells keep the minimum intracellular ATP concentration to ensure survival and that this level is increased in two steps during the recovery phase. Future research using starving cells in similar extreme conditions should further elucidate the physiological function of the β-hairpin structure and its evolutionary significance for phototrophic organisms.

## Experimental procedures

### Materials

ATP, ADP, 9-amino-6-chloro-2-methoxy-acridine (ACMA), carbonyl cyanide 4-(trifluoromethoxy)phenylhydrazone (FCCP), and valinomycin were obtained from ORIENTAL YEAST, Millipore, Invitrogen Life Technologies, Wako, and Sigma Aldrich, respectively. *N*-octyl-β-d-glucopyranoside (OG) and lauryl maltose neopentyl glycol (LMNG) were obtained from Anatrace. Pyruvate kinase, lactate dehydrogenase, and NADH were obtained from Roche Diagnostics. Other chemicals were of the highest commercially available grade.

### Bacterial strain and culture conditions

Cells of the glucose-tolerant strain *S*. 6803 (47) were grown at 30 °C in liquid BG11 medium (48) supplemented with 20 mM Hepes-NaOH (pH 7.5) and bubbled with 1% (v/v) CO_2_-enriched (or ambient) air under continuous illumination with white fluorescent lamps (40 μmol photons m^−2^s^−1^). For antibiotic selection and maintenance of mutant strains, cells were grown on 1.5% (w/v) BG11 agar plates (Bacto Agar, Difco) supplemented with 0.3% (w/v) sodium thiosulfate.

### Construction and transformation of *S.* 6803 mutants

Genetic engineering of *S.* 6803 was performed using the homologous recombination technique. Construction for the addition of a His_10_-tag into the N terminus of the β subunit is described in the supplemental information (Fig. S2). The mutant was selected using the chloramphenicol acetyltransferase gene. Transformants of *S*. 6803 were selected on BG11 plates for chloramphenicol (20 μg ml^−1^) resistance. These mutants (“WT” in the text) were then applied as a parental cell line for further mutagenesis. The deletion of the insertion region of the γ subunit was performed using a plasmid in which *sll1327* was cloned adjacent to the kanamycin-resistance gene (30). A plasmid for the deletion of amino acids 205–220 of the γ subunit (Δ205–220/E204G/K221G, “Δ205–220” in the text) was constructed using the megaprimer PCR method (49). For the first PCR, two primers were used: sll1327_Fw as a forward primer and sll1327_del205_220_Rv as a reverse primer. For the second PCR, slr1411_Rv and the DNA fragment obtained from the first PCR were used as primers. The resultant DNA fragment was cloned into the pGEM-T Easy vector (Promega) according to the manufacturer’s instructions. A plasmid for the deletion of amino acids 212–213 of the γ subunit (Δ212–213/R211G/K214G, “Δ212–213” in the text) was constructed using the quick-change method with PrimeSTAR Max (Takara) according to the manufacturers’ instructions, using primers sll1327_del212_213_Fw and sll1327_del212_213_Rv. Plasmids selected by DNA sequencing were mixed with *S.* 6803 cells, and the transformants were selected and subcultured in the presence of 10 μg ml^−1^ chloramphenicol and 10 μg ml^−1^ kanamycin. The primers used for the preparation of mutants are listed in Table S1.

### Purification of F_o_F_1_ complexes from *S.* 6803

One liter of cells grown under normal conditions (30 °C, 1% CO_2_, continuous light illumination, 40 μmol photons m^−2^s^−1^) was harvested at the late log phase by centrifugation, followed by flash-freezing in liquid nitrogen and storage at −80 °C until use. The cells resuspended in a buffer containing 20 mM Hepes-KOH (pH 8.0), 10 mM NaCl, 0.1 mM MgCl_2_, and 0.1 mM ATP were broken by vortexing with zircon beads and the homogenate was centrifuged for 10 min at 3000*g* at 4 °C to remove cell debris. The supernatant was then centrifuged at 125,000*g* for 30 min at 4 °C to precipitate thylakoid membranes. The membranes were washed with a buffer containing 20 mM Hepes-KOH (pH 8.0), 0.1 mM MgCl_2_, 10% glycerol, and 0.1 mM ATP, followed by solubilization in 1% LMNG for 60 min at 4 °C. Solubilized membrane proteins were obtained after centrifugation at 201,000*g* for 30 min at 4 °C and subjected to Ni-affinity chromatography in a buffer containing 10 mM potassium phosphate, pH 8.0, 100 mM K_2_SO_4_, 0.1 mM MgCl_2_, 0.1 mM ADP, 0.005% LMNG, and 60 to 200 mM imidazole. The obtained F_o_F_1_ preparations were concentrated using Amicon Ultra (Millipore) with a buffer containing 10 mM potassium phosphate, pH 8.0, 100 mM K_2_SO_4_, 0.1 mM MgCl_2_, 0.1 mM ADP, and 0.005% LMNG and then flash-frozen in liquid nitrogen and stored at −80 °C after the addition of glycerol at a final concentration of 10%. The concentration of the purified F_o_F_1_ was determined using Pierce BCA Protein Assay Kit (Thermo Fisher Scientific) with BSA as the protein standard.

### SDS-PAGE, immunoblotting, and N-terminal Edman sequencing

Proteins were separated by sodium dodecyl sulfate–polyacrylamide gel electrophoresis (SDS-PAGE) and stained with Quick CBB (Wako). For immunoblotting, separated proteins were transferred onto a polyvinylidene difluoride (PVDF) membrane (Immun-Blot, Bio-Rad). Antibodies against the *c* subunit were obtained from Agrisera, and those against the β subunit were as described in the literature (31). Chemiluminescence was detected using horseradish peroxidase–conjugated secondary antibodies and ECL Prime (Life Technologies) and visualized on a LAS 3000 mini instrument (GE Healthcare). Images were digitized using the ImageJ software. Otherwise, the separated proteins were transferred onto a membrane (Sequi-Blot PVDF Membrane, Bio-Rad). The N-terminal sequences were determined by Edman degradation on a peptide sequencer (PPSQ21, Shimadzu), based on a previous study (50).

### In-gel digestion and peptide-mapping analysis

Proteins stained with Coomassie Brilliant Blue R-250 were excised from the SDS-PAGE gel and in-gel digested using trypsin. The resulting peptides were analyzed by mass spectrometry as described (50). The parameters used for database searches were as follows: database, Cyanobase_S6803GTI; enzyme, trypsin; fixed modifications, carbamidomethyl (C); variable modifications, oxidation (M); mass values, monoisotopic; peptide mass tolerance, ± 100 ppm; max missed cleavages, 2.

### Reconstitution into PLs

PLs were reconstituted as described (36), with some modifications. Crude soybean l-α-phosphatidylcholine (type II-S; Sigma) was suspended at a final concentration of 32 mg ml^−1^ in Rec-buffer (15 mM MES-Tricine, 2 mM KOH, 5 mM NaCl, 2.5 mM MgCl_2_, and 50 mM sucrose, with the pH adjusted to 8.0 with NaOH). The suspension was incubated for 5 min, followed by brief sonication with a water bath sonicator and centrifugation at 125,000*g* for 30 min at 20 °C. After two or three repetitions of these procedures, the suspension was divided into aliquots, frozen in liquid nitrogen, and stored at −80 °C until use. The reconstitution of F_o_F_1_ into liposomes was performed as follows. The lipid suspension was mixed with an equal volume of Rec-buffer and 2% (w/v) *N*-dodecyl-β-d-glucoside (OG), followed by incubation for 10 min at room temperature. To this solution, 200 mg of Biobeads (SM-2, Bio-Rad) was added until the mixture became unpure. F_o_F_1_ (90 μg) was then added to the solution (final concentration, 0.15 mg ml^−1^). The mixture was incubated at 4 °C overnight, followed by flash-freezing in liquid nitrogen and storage at −80 °C until use.

### ATP hydrolysis/synthesis and proton-translocating activities

ATP hydrolysis activity was measured using an ATP-regenerating system as described (31), with some modifications. The assay was conducted at 30 °C. For measurements of ATP synthesis activity, we applied the acid–base transition method with valinomycin-induced diffusion potential of K^+^, as described (36). The PL suspension (30 μl) was acidified by mixing with 70 μl of an acidic buffer (50 mM MES-Tricine, 2 mM KOH, 50 mM NaCl, 50 mM sucrose, 10 mM NaH_2_PO_4_, and 2.5 mM MgCl_2_, adjusted to pH 5.6 by adding NaOH) supplemented with 0.2 nM valinomycin, 0.5 mM ADP, and 0.01 mM P^1^,P^5^-di(adenosine-5′) pentaphosphate (Ap5A), followed by a 10-min incubation at 30 °C. The mixture was then injected into 900 μl of a basic buffer (300 mM Tricine, 200 mM KOH, 50 mM NaCl, 50 mM sucrose, 10 mM NaH_2_PO_4_, and 2.5 mM MgCl_2_, adjusted to pH 8.8 with NaOH, 0.5 mM ADP, and 0.01 mM Ap5A) supplemented with 100 μl of a luciferin/luciferase-containing solution (ATP Bioluminescence Assay Kit CLS II, Roche Diagnostics). The luminescence was detected by a luminometer (Luminescencer AB2200, ATTO). After 50 s, 5 μl of 10 μM ATP was added three times, for calibration. The initial rate of ATP synthesis was calculated from the exponential fit of the initial 0 to 45 s after the injection of the acidified PLs. ATP-driven proton-translocating activity was measured using a fluorometer (FP8500, Jasco) and fluorescence quenching of ACMA (excitation at 410 nm, emission at 480 nm) at 30 °C, as described (51). PLs (50 μl; final concentration, 6.25 μg ml^−1^) were injected into a buffer containing 15 mM MES-Tricine, 2 mM KOH, 5 mM NaCl, 2.5 mM MgCl_2_, and 50 mM sucrose, adjusted to pH 8.0 with NaOH and 0.3 μg ml^−1^ ACMA. The reaction was initiated by adding 2 mM ATP. When indicated, 1 μg ml^−1^ FCCP was added.

### Measurements of intracellular chlorophyll content, cell density, and absorption spectra

For the extraction of chlorophylls, cells were suspended in 100% methanol, followed by sonication and centrifugation at 20,000*g* for 10 min, to precipitate cell debris. Chlorophyll content (μg ml^−1^) was calculated from the following equation: chlorophyll content = 13.4 × *A*_665_. Cell density was monitored as absorbance at 750 nm (*A*_750_) on a spectrophotometer (UV-1800, Shimadzu). For measurements of cell absorption spectra, cells were collected by centrifugation and resuspended in BG11 medium (*A*_750_ = 1.0), followed by measurements using a spectrophotometer equipped with an integrating sphere (V-650, Jasco).

### Microscopic analysis

Bright-field microscopic analysis of cells grown under continuous light conditions (40 μmol photons m^−2^ s^−1^) was performed using a PlanApo N 60×/1.45 oil objective fitted on an Olympus IX73 microscope.

### Intracellular ATP/ADP level determination

Intracellular ATP/ADP level was determined according to the literature (30), with some modifications. Cell culture (100 μl) was withdrawn and added to 20 μl of 12% perchloric acid. After incubation on ice for at least 30 min, the solution was centrifuged at 20,000*g* for 10 min at 4 °C to precipitate cell debris. Subsequently, 100 μl of the supernatant was neutralized with 200 μl of 2 M Tris-acetate, pH 7.7. The levels of ATP or ATP + ADP were quantified after incubation for 3 h at 25 °C in the absence or presence of pyruvate kinase in a buffer (10 mM Tris–acetate, pH 7.7, 10 mM KCl, 1 mM MgCl_2_, ±10 mM phosphoenolpyruvate, and ±57 μg ml^−1^ pyruvate kinase). The luminescence was quantified using CLSII (Sigma-Aldrich) and a luminometer, Tristar (Berthold Technologies).

### Quantification of intracellular glycogen content

Glycogen was quantified based on a previous study (52). Cells were suspended in 500 μl of 3.5% (v/v) sulfuric acid and boiled at 100 °C for 120 min, followed by centrifugation at 20,000*g* for 10 min. Then, 6.7 μl of supernatant was mixed with 1 ml of the reaction mixture of LabAssay Glucose (Wako), followed by measurements according to the manufacturer’s instructions.

## Data availability

All data are contained within the article and can be shared upon request (thisabor@res.titech.ac.jp).

## Supporting information

This article contains supporting information (31, 53).

## Conflict of interest

The authors declare that they have no conflicts of interest with the contents of this article.
