# Supplementary material for: The phototroph-specific β-hairpin structure of the γ subunit of FoF1-ATP synthase is important for efficient ATP synthesis of cyanobacteria
Source: J Biol Chem. 2021 Jul 31;297(3):101027. doi: 10.1016/j.jbc.2021.101027 (PMC8390522; doi:10.1016/j.jbc.2021.101027)
Supplement: Supplemental Figures S1–S6 and Table S1 [file mmc1.pdf]

Supplementary information

Supplementary methods

Extraction of crude F_1 fraction

1 L of cells grown under the normal conditions (30°C, 1% CO_2, continuous light illumination, 40 µmol photons m^{-2} s^{-1}) were harvested at the late log phase by centrifugation, followed by flash-frozen in liquid nitrogen and stored at -80°C until use. The cells resuspended in a buffer containing 20 mM *N*-2-hydroxyl piperazine-*N'*-2-ethane sulfonic acid (HEPES)-KOH (pH 8.0), 10 mM NaCl, 0.1 mM $MgCl_2$, and 0.1 mM ATP were broken by vortexing with zircon beads, and the homogenate was centrifuged for 10 min at 3,000 × *g* at 4°C to remove cell debris. The supernatant was then centrifuged at 125,000 × *g* for 30 min at 4°C to precipitate thylakoid membranes. The membranes were resuspended in a buffer containing 0.5 M sucrose, 4 mM EDTA, 40 mM Tricine-KOH, pH 8.0, 0.1 mM ATP, followed by incubation for 60 min at room temperature, to remove F_1 from thylakoids. Membrane proteins were precipitated by mixing with a one-half volume of chloroform and the following centrifugation at 125,000 × *g* for 30 min at 20°C. The supernatant was diluted with ten-volume of a buffer containing 20 mM potassium phosphate (pH 8.0), 100 mM K_2SO_4, and 0.1 mM ATP, followed by Ni-affinity chromatography using Ni Sepharose 6 Fast Flow (Cytiva, Marlborough, MA, USA). Obtained F_1 complexes concentrated using Amicon Ultra (Millipore) with a buffer, containing 50 mM HEPES-KOH (pH 8.0), 100 mM KCl, 0.1 mM $MgCl_2$, and 0.1mM ATP were flash-frozen in liquid nitrogen and stored at -80ºC after the addition of glycerol at a final concentration of 10% (v/v). Protein concentrations were determined using the BCA method (Bio-Rad Protein Assay, Bio-Rad Laboratories, Inc., Hercules, CA, USA).

Measurements of ATP hydrolysis activities with/without LDAO

ATP hydrolysis activity was measured using an ATP-regenerating system as described previously (31), with some modifications. The assay was conducted at 30°C. When indicated, LDAO was added to the reaction mixture at a final concentration of 0.1%.

Photosynthetic activity measurements

Chlorophyll fluorescence was measured using Dual-PAM 100 (Walz, Germany). Before the measurements, *S.* 6803 cells were adjusted to OD_{750} = 0.5. The minimum chlorophyll fluorescence (Fo) was measured after the dark-acclimation for 20 min. Actinic light treatment (30 µmol photons m^{-2} s^{-1}) was then delivered for 20 min to obtain the fluorescence parameters, steady-state fluorescence (F), and maximum fluorescence in the light (Fm'). After switching off the actinic light, 20 µM 3-(3,4-dichlorophenyl)-1,1-dimethylurea (DCMU) was added, and the maximum chlorophyll fluorescence (Fm) was determined after reirradiation of the actinic light. The photosynthetic parameter was calculated using the following equation: $\Phi II = (Fm' - F)/Fm'$ (53).

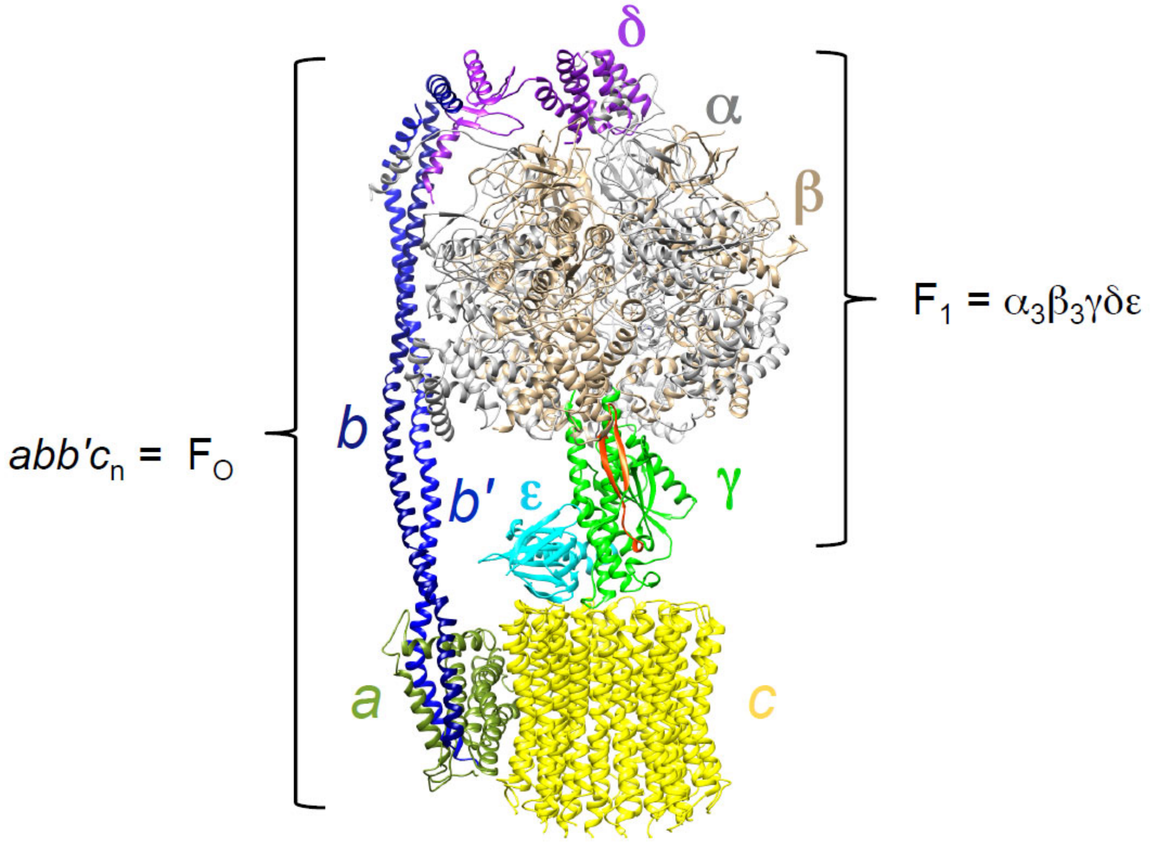

Fig. S1. A schematic diagram of F_oF_1. The γ and ε subunits from *T. elongatus* (PDB ID: 5ZWL) were superimposed on those from *S. oleracea* (PDB ID: 6VOH). The γ and ε subunits from *S. oleracea* were removed for clarity. The green and orange-red colors indicate the γ subunit and the β-hairpin structure, respectively. The gray, beige, sky-blue, and purple colors indicate the α, β, ε, and δ subunits, constituting a hydrophilic rotary motor, F_1. The olive, navy-blue, blue, and yellow colors indicate the *a, b, b′,* and *c* subunits, which constitute an intramembrane rotary motor, F_o.

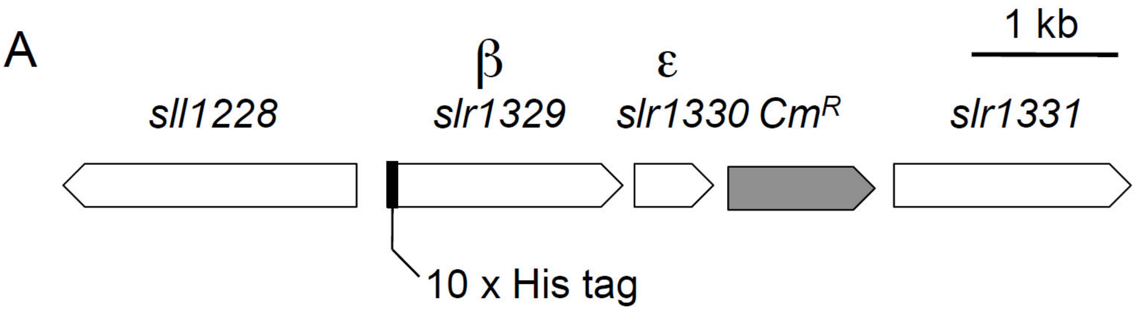

B

```
AAACCTCCCCCCGGCGATCGCCTATCCTCCTAGGATTTGACCCATCTCACCAGTTGGGGACAGCCCCAAAGTGATACCCTT
AAAGATGGCAAATTTGACCAAAACTCAACAGTAATCCTGTAGTCTAACTATAAACAAATTCCTAGACAATCAGCATGCATC
ACCATCATCACCATCACCATCACCATATGGTAGCCGTAAAAGAAGCAACTAACGTTGGCAAAATTACCCAGGTCATCGGGC
CTGTAATTGACGCCCAGTTCCCCAGTGGTAAATTGCCCCGTATTTATAATGCCCTTAAAGTCCAAGGCAGAAACTCTGCTG
GTAACGAAGTAGCTGTTACCTGTGAAGTGCAGCAGCTTCTCGGCGATAACCAAGTCCGAGCCGTAGCCATGAGTTCCACCG
ACGGTCTCGTCCGGGGCATGGACGTGGTAGACACCGGGGCCCCCATCAGCGTTCCCGTCGGCACCGGCACCCTGGGTCGTA
TTTTTAACGTTCTTGGTGAGCCTGTTGACAACAAAGGCCCCGTGCCCGCTGGTGAAACTTTCCCCATTCACCGTCCCGCTC
CCAAATTGGTGGATTTGGAAACCAAGCCCCAAGTATTTGAAACCGGCATTAAGGTAATTGACCTGCTTACTCCCTACCGTC
AGGGTGGCAAAATCGGTCTCTTCGGTGGTGCTGGTGTGGGCAAAACCGTAATCATGATGGAATTGATTAACAACATCGCCA
TCCAACATGGTGGTGTATCTGTATTTGGTGGCGTAGGGGAACGGACCCGGGAAGGGAATGACCTCTACAACGAAATGATCG
AATCCAACGTAATCAACGCCGACAAACCGGAAGAGTCCAAAATTGCTCTGGTGTACGGTCAGATGAACGAACCCCCCGGGG
CTCGGATGCGGGTAGGCTTAACCGCTTTGACCATGGCGGAATATTTCCGGGATGTGAACAAACAGGACGTATTGCTCTTCA
TCGACAACATTTTCCGCTTCGTCCAAGCTGGTTCGGAAGTATCGGCTCTGTTGGGCCGGATGCCCTCTGCGGTAGGTTACC
AGCCCACTTTAGGTACGGACGTTGGTGATTTGCAAGAGCGTATCACCTCCACCAAGGAAGGTTCCATTACCTCCATTCAGG
CTGTGTATGTACCGGCGGACGACTTGACTGACCCCGCCCCCGCCACCACCTTTGCCCACTTGGACGGTACCACCGTGCTTT
CCCGTGGTTTGGCCGCTAAAGGTATTTACCCCGCCGTGGACCCCTTGGATTCCACCAGCACCATGCTTCAGCCCTCCATCG
TTGGGTCAGAGCATTACGACACCGCTCGGGAAGTGCAATCCACCCTGCAACGCTACAAAGAATTGCAAGATATTATTGCCA
TTCTTGGCTTGGATGAATTGTCTGAGGAAGACCGTTTGACCGTAGACCGGGCTCGGAAAATTGAGCGTTTCCTTTCCCAAC
CCTTCTTCGTCGCCGAAGTATTTACCGGTGCCCCCGGCAAGTACGTTTCCCTGGCTGACACCATCAAAGGT
```

Fig. S2. Construction of the His-tag fused β subunit of *S.* 6803. *A*, a schematic diagram of the gene arrangement expected in the strain, expressing His-tagged β subunit ('WT' in the text). The position of the N-terminal His x 10 tag was indicated in a black box. *B*, a result of DNA sequence. PCR products obtained by using the 'WT' genome as a template and the primers, 5'-TCGCAATTGCCCGCAGACAATCTT-3' and 5'-GCTCCGGTAAATCATCCAATTCAC-3', were applied for DNA sequence analysis using the same primers. The sequence encoding His-tag and the β subunit were colored in red and blue, respectively.

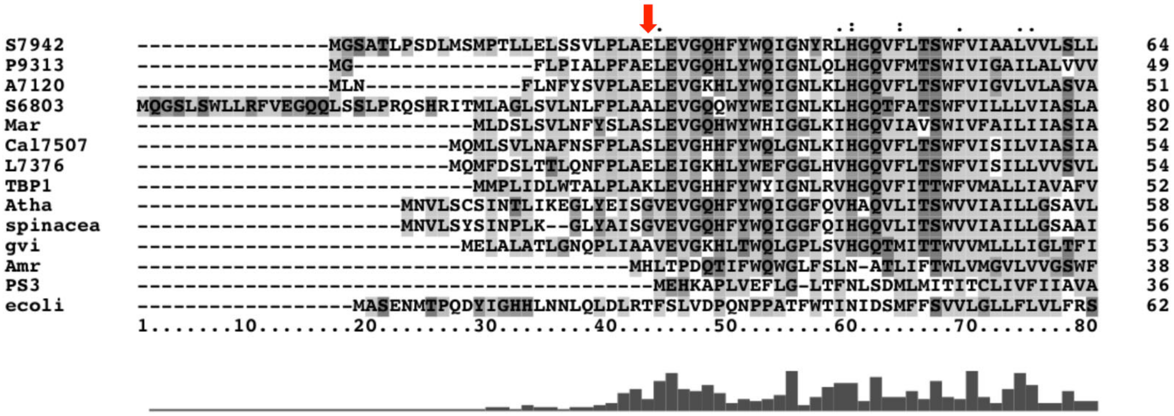

Fig. S3. N-terminal amino-acid sequence alignment of the *a* subunit of F_oF_1. N-temrinal amino-acid sequences of the a subunits from *Synechococcus elongatus sp.* PCC 7942 (S7942), *Prochlorococcus marinus* MIT 9313 (P9313), *Anabaena* sp. PCC 7120 (A7120), *S.* 6803 (S6803), *Microcystis aeruginosa* (Mar), *Calothrix* sp. PCC 7507 (Cal7507), *Leptolyngbya* sp. PCC 7376 (L7376), *T. elongatus* (TBP1), *Arabidopsis thaliana* (Atha), *Spinacia oleracea* (spinacea), *Gloeobacter violaceus* (gvi), *Acaryochloris marina* (Amr), *Bacillus* sp. PS3 (PS3), *Escherichia coli* (ecoli) were compared. The alignment was generated with the Clustal W multiple alignment tool. A red arrow indicates the sequenced N-terminus in *S.* 6803. Asterisks and dots indicate highly conserved amino acids.

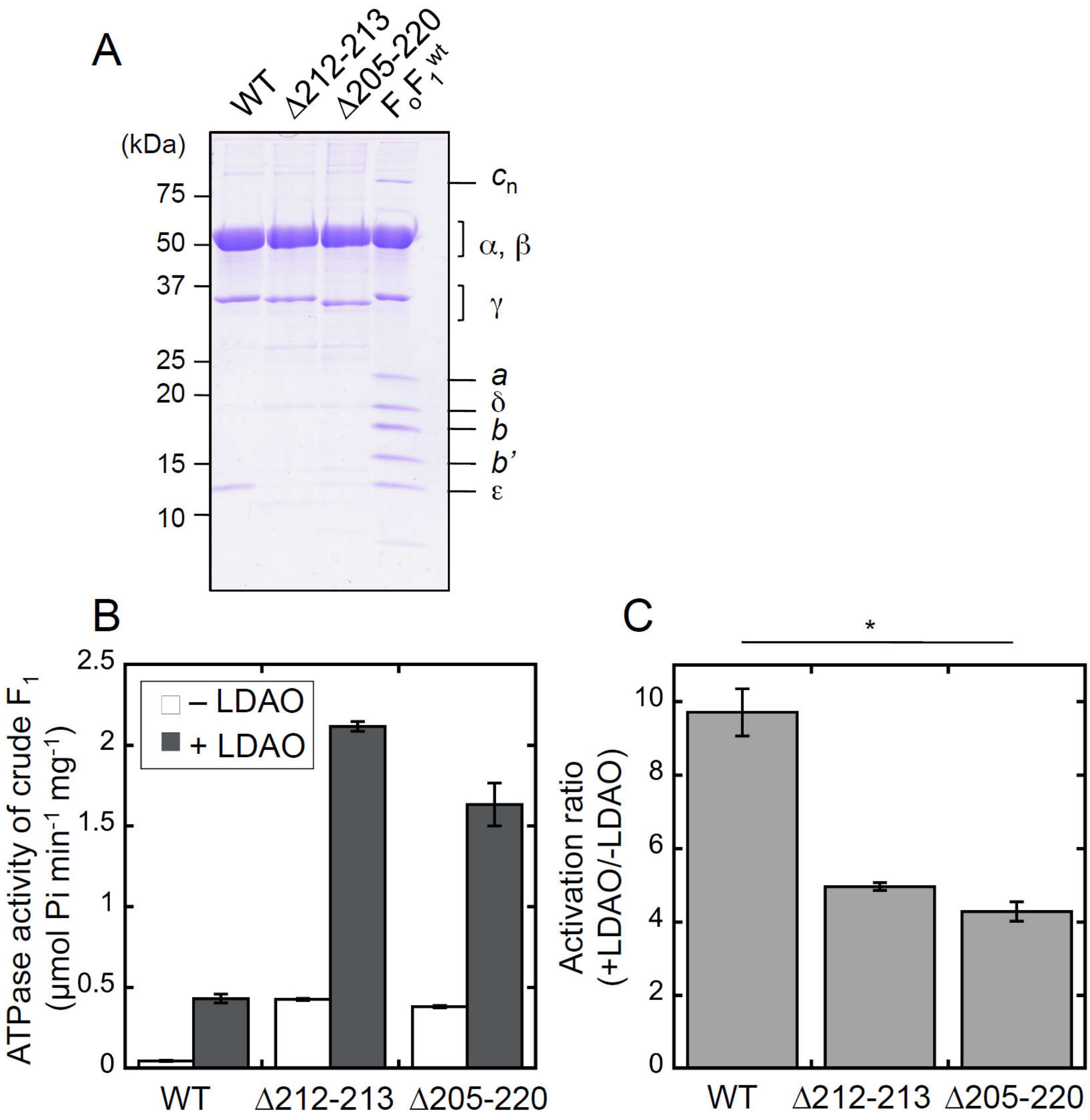

Fig. S4. Effect of LDAO on ATP-hydrolysis activity of crude F_1 fraction. *A*, crude F_1 fractions were obtained by nickel affinity chromatography after chloroform extraction. Wild-type F_oF_1 was loaded as a control ($F_oF_1^{wt}$). A total of 5 μg of proteins was loaded per lane. *B*, ATP-hydrolysis activities were measured using the ATP-regenerating system. The assay was conducted at 30°C. The activity was determined from the slope at the steady-state. White and black bars indicate the activities in the absence and presence of LDAO (final concentration of 0.1%), a nonionic detergent, respectively. Results of 3-5 independent experiments were averaged (mean ± SD). *C*, the extents of the activation by the addition of LDAO were calculated from the results shown in *B*. The asterisk indicates statistical significance ($P < 0.05$, Welch's t-test).

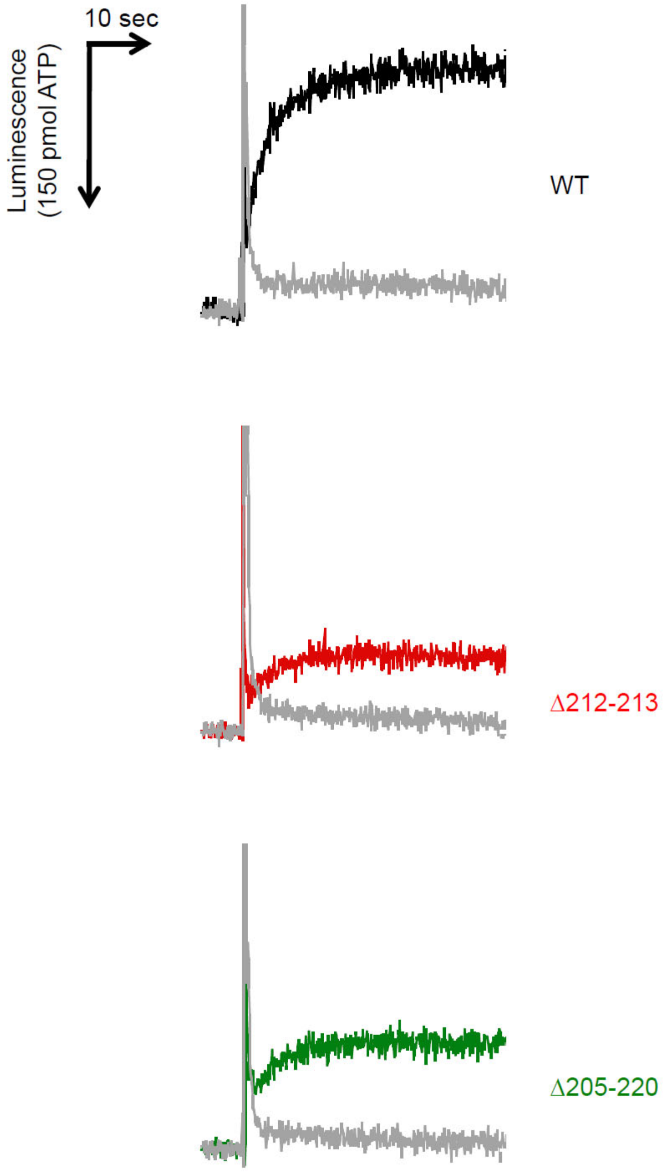

Fig. S5. Sensitivity of ATP-synthesis activity to the uncoupler nigericin. ATP synthesis activities of PLs were measured as described in Fig. 5. Nigericin was added prior to the measurements (500 nM, gray traces) to check if each activity was derived from ΔpH-driven ATP synthesis.

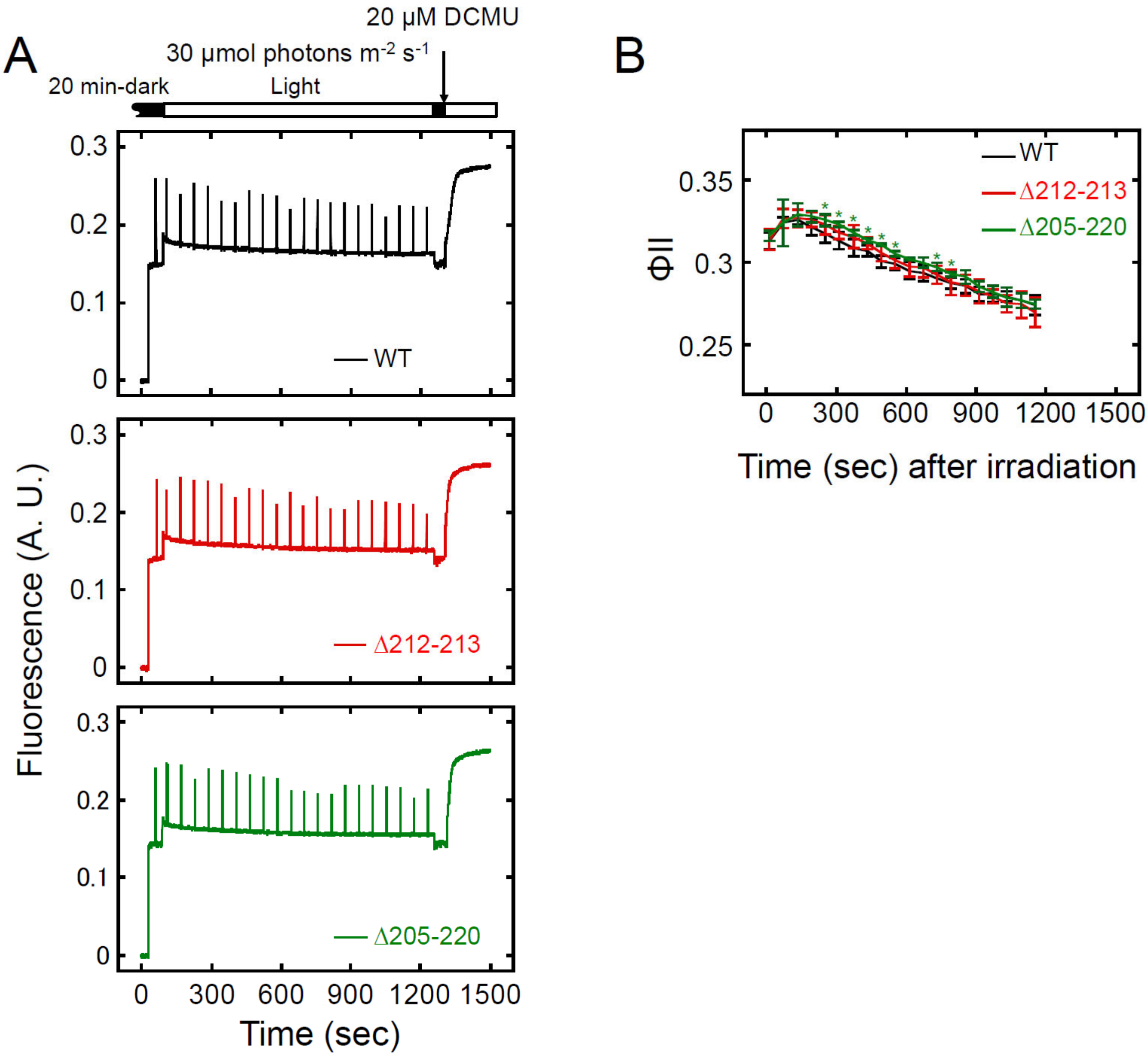

Fig. S6. The photosynthesis activities of *S.* 6803 measured by chlorophyll fluorescence. *A*, pulse amplitude modulation fluorescence analysis of WT (black trace), Δ212-213 (red trace), and Δ205-220 (green trace). Before the measurements, cells were dark-acclimated for 20 min. The saturating pulses were then applied every 60 sec during the actinic light irradiation (Light). At the end of each measurement, DCMU (20 µM) was added to obtain the maximum fluorescence. *B*, the effective PSII quantum yield (ΦII) during the actinic-light illumination. The X-axis indicates the time (seconds) after actinic light exposure. Data are the mean ± SD from three or four independent experiments. The asterisks indicate statistical significance ($P < 0.05$, Welch's t-test).
